# Hydrogenase-3 Contributes to Anaerobic Acid Resistance of *Escherichia coli*

**DOI:** 10.1371/journal.pone.0010132

**Published:** 2010-04-12

**Authors:** Ken Noguchi, Daniel P. Riggins, Khalid C. Eldahan, Ryan D. Kitko, Joan L. Slonczewski

**Affiliations:** Department of Biology, Kenyon College, Gambier, Ohio, United States of America; Auburn University, United States of America

## Abstract

**Background:**

Hydrogen production by fermenting bacteria such as *Escherichia coli* offers a potential source of hydrogen biofuel. Because H_2_ production involves consumption of 2H^+^, hydrogenase expression is likely to involve pH response and regulation. Hydrogenase consumption of protons in *E. coli* has been implicated in acid resistance, the ability to survive exposure to acid levels (pH 2–2.5) that are three pH units lower than the pH limit of growth (pH 5–6). Enhanced survival in acid enables a larger infective inoculum to pass through the stomach and colonize the intestine. Most acid resistance mechanisms have been defined using aerobic cultures, but the use of anaerobic cultures will reveal novel acid resistance mechanisms.

**Methods and Principal Findings:**

We analyzed the pH regulation of bacterial hydrogenases in live cultures of *E. coli* K-12 W3110. During anaerobic growth in the range of pH 5 to 6.5, *E. coli* expresses three hydrogenase isoenzymes that reversibly oxidize H_2_ to 2H^+^. Anoxic conditions were used to determine which of the hydrogenase complexes contribute to acid resistance, measured as the survival of cultures grown at pH 5.5 without aeration and exposed for 2 hours at pH 2 or at pH 2.5. Survival of all strains in extreme acid was significantly lower in low oxygen than for aerated cultures. Deletion of *hyc* (Hyd-3) decreased anoxic acid survival 3-fold at pH 2.5, and 20-fold at pH 2, but had no effect on acid survival with aeration. Deletion of *hyb* (Hyd-2) did not significantly affect acid survival. The pH-dependence of H_2_ production and consumption was tested using a H_2_-specific Clark-type electrode. Hyd-3-dependent H_2_ production was increased 70-fold from pH 6.5 to 5.5, whereas Hyd-2-dependent H_2_ consumption was maximal at alkaline pH. H_2_ production, was unaffected by a shift in external or internal pH. H_2_ production was associated with *hycE* expression levels as a function of external pH.

**Conclusions:**

Anaerobic growing cultures of *E. coli* generate H_2_ via Hyd-3 at low external pH, and consume H_2_ via Hyd-2 at high external pH. Hyd-3 proton conversion to H_2_ is required for acid resistance in anaerobic cultures of *E. coli*.

## Introduction

Bacterial hydrogen production by hydrogenase is studied as a promising source of clean alternative energy [1], [2]. In the intestinal tract, H_2_ produced from bacteria fermentation enables methane production by methanogens [3] and contributes to the growth of pathogens such as *Salmonella enterica* and *Helicobacter pylori* [4], [5]. Hydrogenase in *Escherichia coli* has been suggested to decrease cytoplasmic acid stress and contribute to its acid resistance systems [6]–[9]. Because *E. coli* need to survive the harshly acidic environment of the stomach to colonize the intestine, acid resistance systems enhance the infective ability of pathogenic *E. coli* [10]–[12].

Several mechanisms have been characterized that enhance survival at pH 2.5 and below [13], such as the amino acid-dependent glutamate and arginine decarboxylases [14]–[16]. Genes encoding these enzymes and transporters are up-regulated during growth in moderate acid [7], [17], [18]. Most of the above studies of *E. coli* acid resistance address aerated cultures. In natural environments such as the gastrointestinal tract, however, enteric bacteria experience low oxygen. Oxygen limitation and acid stress occur in the microaerobic environment of the stomach [19], which harbors many obligate and facultative anaerobic organisms such as *Clostridium* and *Veillonella* species [20], [21]. In *Salmonella typhimurium,* anoxic conditions are required for expression of the acid-resistance component arginine decarboxylase [22]. Hayes *et al.* (2006) showed that all four hydrogenase isoenzymes are upregulated by acid under oxygen-limited conditions [7].

The four isoforms of hydrogenase catalyze the reversible oxidation of molecular hydrogen to 2H^+^. However, each hydrogenase functions primarily in one direction. Hydrogenase-1 (Hyd-1, encoded by *hya*) and hydrogenase-2 (Hyd-2, encoded by *hyb*) are energy-conserving respiratory pathways consuming H_2_ with Hyd-2 acting as the primary consumption hydrogenase [23]–[25]. Hydrogenase-3 (Hyd-3) is the primary production hydrogenase [26]; along with formate dehydrogenase (FDH-H), Hyd-3 makes up the formate hydrogen lyase (FHL) complex, which breaks down formate to carbon dioxide and H_2_ [6], [27]–[29]. The function of hydrogenase-4 (Hyd-4) is unclear; it may be a component of a second formate hydrogen lyase system (FHL-2) [26], [30], [31]. Mutants deleted for *hypF* mutant lacks all hydrogenase activity [8], [32]. A *hypF* mutant shows decreased acid resistance in partly aerated cultures [7].

Because the function of hydrogenases is intricately connected to metabolic pathways, the pH-dependence of H_2_ consumption must be measured *in vivo*. Previous studies of H_2_ production of hydrogenase mutants have been based on harvested cell concentrates, often with addition of 100 mM formate to increase FHL activity, although such high formate concentration is incompatible with growth [9], [25], [33]. Our electrode-based methods were applied to live, growing cultures. Since H_2_ production was hypothesized as a cellular mechanism for acid resistance, we observed the pH-dependent activity of hydrogenases under the conditions where acid resistance is induced (anaerobic growth at low external pH). We also characterized how pH regulates H_2_ production and consumption via each hydrogenase complex, determining the significance of each at low and high pH.

## Results

### H_2_ production as a function of pH

**Figure 1:**
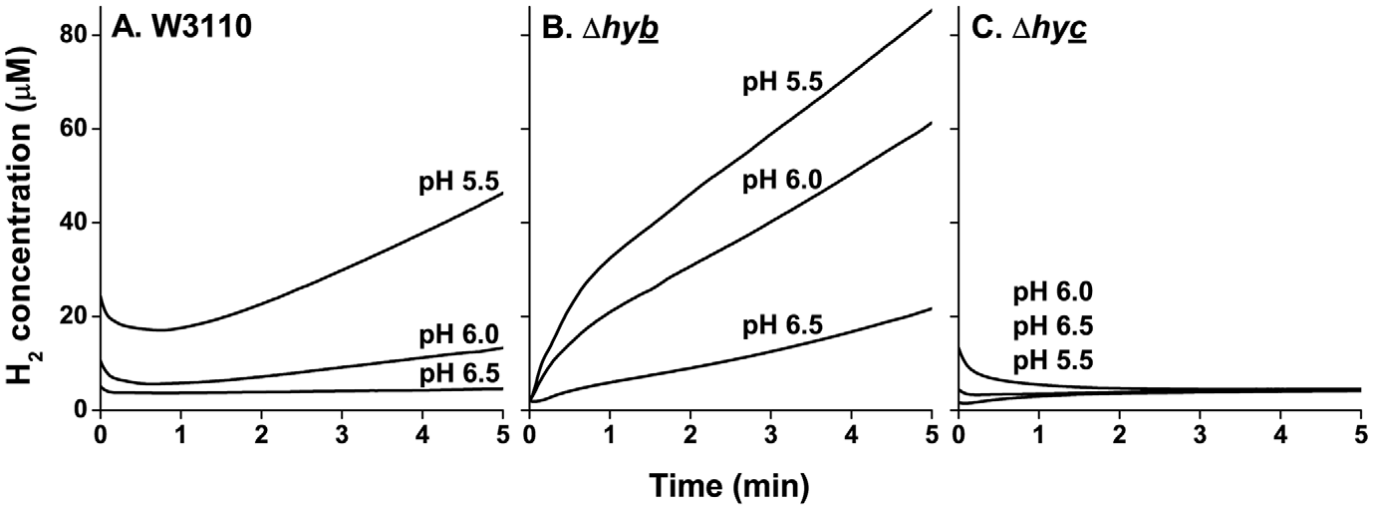
Effect of pH on the H_2_ production by W3110, Δ*hybC*, and Δ*hycE*. The lines represent traces of H_2_ concentration as a function of time. Before time 0, the cultures were sparged with 100% N_2_ in order to eliminate any residual H_2_ in the culture. Anaerobic cultures of W3110 (**A**), Δ*hybC* (**B**), and Δ*hycE* (**C**) were grown to log phase at pH 5.5, pH 6, and pH 6.5 and assayed for H_2_ production as stated in the Materials and Methods. Lines are representative samples of n = 3.

In order to test the role of hydrogenases at low pH, we observed *E. coli* H_2_ production across a range of pH values in strains W3110, JLS0920 (lacks Hyd-1), JLS0921 (lacks Hyd-2), and JLS0922 (lacks Hyd-3). H_2_ levels were measured using a Unisense electrode, as described under Materials & Methods. Our results using strain W3110 show that H_2_ production increased as pH decreased (Fig. 1). In strain JLS0921, lacking the primary consumption hydrogenase (Hyd-2), H_2_ gas was produced without consumption. In this strain a similar pattern of increasing H_2_ production with decreasing pH was observed (Fig. 1B), and at each pH H_2_ was produced faster than in the parental strain (Fig. 1A). By contrast, strain JLS0922, lacking the primary production hydrogenase Hyd-3, showed virtually no H_2_ production at any pH (Fig. 1C). Strain JLS0920, lacking Hyd-1, showed no difference in H_2_ production from the wild-type (data not shown).

**Figure 2:**
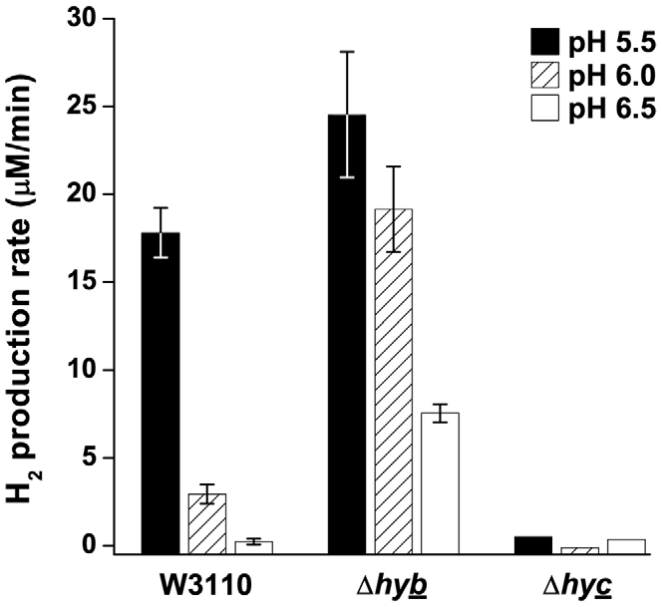
Effect of pH on the H_2_ production rate of W3110, Δ*hybC,* and Δ*hycE*. Anaerobic cultures were grown to log phase at pH 5.5 (black bars), 6 (hatched bars), and 6.5 (white bars) and assayed for H_2_ production as described in the Materials and Methods. H_2_ production rate was calculated as stated in the Materials and Methods. Error bars represent SEM, n = 3; those that were too small to see clearly were omitted. The experiment was conducted twice.

The traces of H_2_ concentration (µM) over time were converted into production rates by taking the slope from 2–5 minutes (Fig. 2). In strain W3110, H_2_ production rate increased sharply at pH 5.5, showing a 70-fold increase from external pH 6.5 to 5.5. Meanwhile, above pH 6.5 H_2_ production was not detected (data not shown). Overall, conversion of protons to H_2_ by Hyd-3 was greatly increased at acidic pH.

In order to determine whether the change in H_2_ production was due to a change in enzymatic activity, W3110 was assayed for H_2_ production and then shifted to alternate pH values. Cells grown to mid-late log phase at pH 5.5 were assayed for 6 minutes before shifting the external pH to 6.5 and cells grown at pH 6.5 were shifted to external pH 5.5. Neither shift changed the H_2_ production (data not shown). Additionally, to assess whether a change in internal pH would affect Hyd-3 activity, benzoate was added to depress cytoplasmic pH. Benzoate is a permeant weak acid that partly equilibrates internal and external pH by dissociating to release protons in the cytoplasm. At external pH 5.5, 5 mM benzoate lowers the cytoplasmic pH from approximately pH 7.5 to pH 6 [34]. Benzoate did not affect H_2_ production at pH 5.5 or pH 6.5 during the 6 min observation after addition (data not shown). Neither changes in external pH nor cytoplasmic pH affected H_2_ production, so the change in H_2_ production was not caused by a change in enzymatic activity.

### *hycE* expression as a function of pH

**Figure 3:**
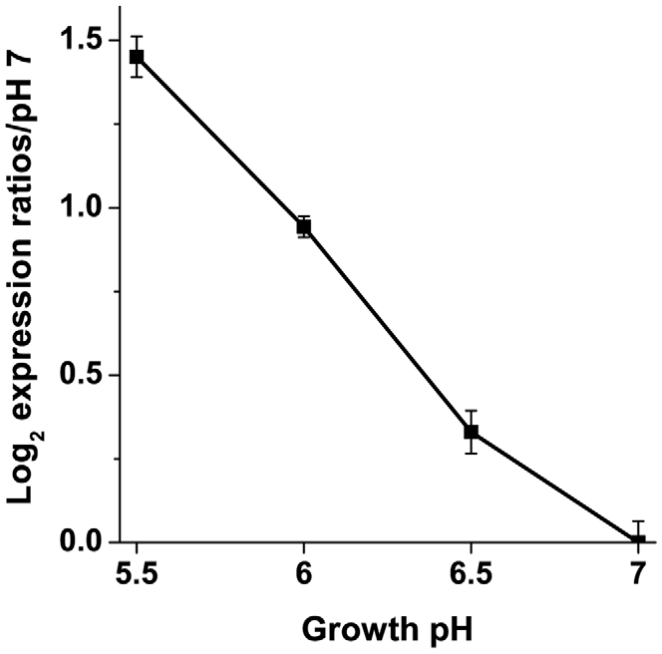
*hycE* gene expression in W3110. RNA was isolated from anaerobic cultures grown to log phase at pH 5.5, 6, 6.5, and 7. Real-Time PCR was used to measure the mRNA levels of *hycE*, the large subunit of Hyd-3. Expression levels were normalized to the pH 7 control. Error bars represent SEM, n = 3 (RNA from independent cultures). The experiment was conducted twice.

In order to determine whether the change in H_2_ production was associated with a change in *hyc* expression, the mRNA levels of *hycE,* encoding the large subunit of Hyd-3, were measured in W3110 from external pH 7 to pH 5.5 (Fig. 3). *hycE* expression increased as the external pH decreased, whereas the activity of Hyd-3 was unchanged with a shift in external or internal pH. Thus the change in H_2_ production more likely arises from the increased *hyc* expression rather than a change in enzyme activity.

### H_2_ consumption as a function of pH

**Figure 4:**
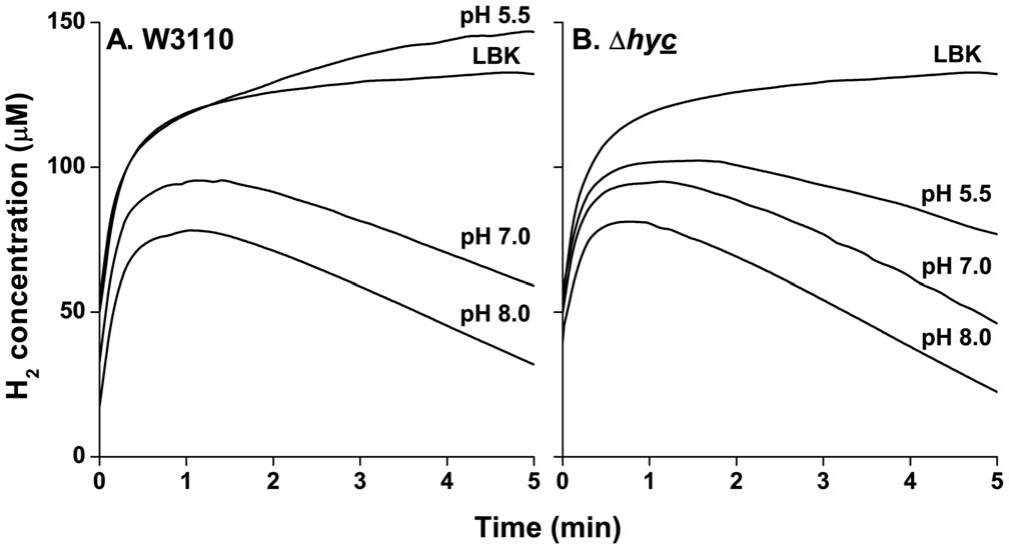
Effect of pH on the H_2_ consumption by W3110 and Δ*hycE*. The lines represent traces of H_2_ concentration as a function of time. Before time 0 the cultures were sparged with 20% H_2_/80% N_2_ in order to saturate the culture with H_2_. Anaerobic cultures of W3110 (**A**) and Δ*hycE* (**B**) were grown to log phase at pH 5.5, pH 7, and pH 8 and assayed for H_2_ consumption as stated in the Materials and Methods. A sample of LBK saturated with H_2_ was assayed as a control to assess residual H_2_ loss. Lines are representative samples of n = 3.

**Figure 5:**
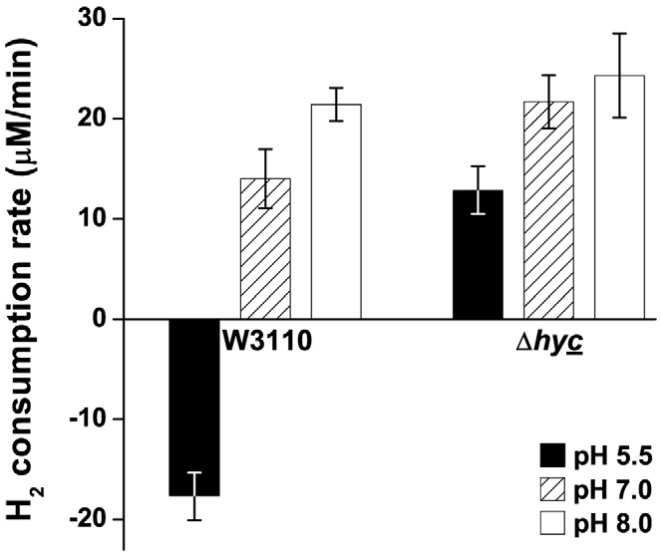
Effect of pH on the H_2_ consumption rate of W3110 and Δ*hycE*. Anaerobic cultures were grown to log phase under anaerobic conditions at pH 5.5 (black bars), 7 (hatched bars), and 8 (white bars) and assayed for H_2_ consumption as described in the Materials and Methods. A negative value for H_2_ consumption means H_2_ is produced. H_2_ consumption rate was calculated as stated in the Materials and Methods. Error bars represent SEM, n = 3. The experiment was conducted twice.

The pH-dependence of H_2_ consumption has yet to be defined. We observed H_2_ consumption in the strains W3110, JLS0920, JLS0921, and JLS0922, cultured at both low and high pH. H_2_ levels were measured as described. Our results using W3110 showed that H_2_ is produced at acidic pH, but at neutral and alkaline pH, H_2_ is consumed (Fig. 4). JLS0920, lacking Hyd-1, showed no difference in H_2_ consumption when compared with the wild-type (data not shown). In JLS0922, lacking Hyd-3, H_2_ was consumed without production. At neutral and alkaline pH, H_2_ production in JLS0922 (Fig. 4B) resembled that observed in the wild-type strain (Fig. 4A), but at pH 5.5 the strain showed only H_2_ consumption. Even in a solution saturated with 20% H_2_, only H_2_ production was observed in JLS0921, lacking Hyd-2 (data not shown). The traces of H_2_ concentration (mV) over time were converted into rates taking the slope from 2–5 minutes (Fig. 5). JLS0922 shows less than a 2-fold difference in consumption from pH 8 to 5.5, suggesting that the regulation of H_2_ consumption is not as strongly pH-dependent as H_2_ production.

### Extreme acid survival of hydrogenase mutants

**Figure 6:**
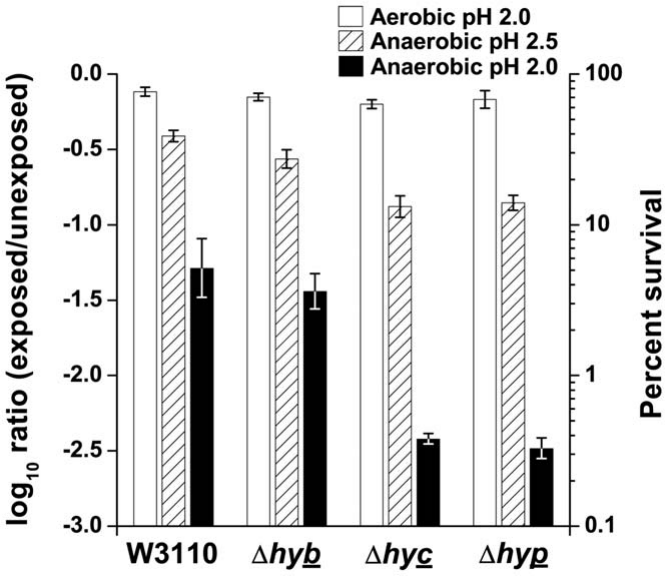
Effect of aeration on the extreme acid survival of W3110, Δ*hybC*, Δ*hycE*, and Δ*hypF*. The white bars represent cultures grown with aeration to stationary phase in LBK buffered at pH 5 that were diluted 200-fold into LBK pH 2 and exposed for 2 h with aeration at 37°C. The hatched and black bars represent anaerobic cultures grown to stationary phase in LBK buffered at pH 5.5 that were diluted 200-fold into LBK pH 2.5 (hatched) or pH 2 (black) and exposed for 2 h without aeration at 37°C. Aerobic and anaerobic cultures were maintained as stated in the Materials and Methods. Error bars represent SEM, n = 5 or 6.

We investigated whether *E. coli* strains grown under conditions of high hydrogenase activity would show hydrogenase-dependent acid resistance. For control comparison, bacteria were cultured aerobically (hydrogenase repressed) to stationary phase at pH 5 and exposed with rotary aeration for 2 h in LBK pH 2 (Fig. 6, white bars). All three hydrogenase mutants, JLS0921, JLS0922, and JLS0925 (lacks all four hydrogenases), showed comparable survival to the wild-type. Similar results were observed with cultures grown aerobically to stationary phase at pH 5.5 (data not shown). Thus, acid survival in aerobic cultures did not require hydrogenase activity.

We then tested the ability of hydrogenase mutants to survive anaerobic acid exposure. Bacteria were grown anaerobically to stationary phase at pH 5.5, and exposed to pH 2.5 (Fig. 6, hatched bars). W3110 and JLS0921 showed comparable survival, but JLS0922 and JLS0925 showed 3-fold decreased acid survival. Survival was also tested at a more extreme acidic condition, pH 2 (black bars). Every strain showed decreased acid survival, but an even larger difference was seen in the acid sensitive strains. JLS0922 (lacking Hyd-3) and JLS0925 (lacking all hydrogenases) showed a 20-fold loss in survival when compared to the wild-type. Thus, Hyd-3 is necessary for extreme acid survival under anoxic conditions, but Hyd-2 is not.

## Discussion

*E. coli* use several mechanisms to resist the harshly acidic conditions of the stomach. A previous report revealed the contribution of hydrogenase to acid resistance in partly aerated cultures [7]. Here we clarify that finding to show that specifically Hyd-3, which is a part of the FHL complex, consumes protons to contribute to acid resistance of anaerobic cultures.

We first defined the pH regulation of both H_2_ consumption and production in various hydrogenase mutants in order to determine which was more important under acidic conditions. Hydrogen production and consumption were measured in vivo, in growing cultures. At acidic pH, H_2_ production was dependent only on Hyd-3, and was increased by the deletion of Hyd-2, whereas H_2_ consumption was only dependent on Hyd-2.

Both H_2_ production and consumption rates are commonly measured under conditions such as cells cultured at pH 6.8 and assayed in the presence of 100 mM sodium formate [25]. Under physiological conditions, intracellular formate concentration reaches only as high as 20 mM [35]. Our results are consistent with the previous finding that Hyd-3 is the main production hydrogenase. We further show that hydrogen production is induced at low pH in the absence of exogenous formate.

The *in vitro* pH-dependent activity of the consumption hydrogenase, Hyd-2, is maximal at high pH [23]. In the current report, we saturated an anaerobic culture with H_2_ to directly measure Hyd-2-dependent H_2_ consumption. This revealed that Hyd-2-dependent consumption increased under alkaline conditions, reaching a maximum at pH 8. Additionally, at pH 5.5 the wild-type strain showed net H_2_ production despite being in a H_2_ saturated environment. Because alkaline conditions appear to enhance H_2_ consumption, it was not expected to contribute to extreme acid survival.

A previous study using *E. coli* MC4100 finds less than 2-fold increase in H_2_ production from pH 7.5 to pH 5.5 [26], whereas we found a 70-fold increase from pH 6.5 to pH 5.5. It is possible that this discrepancy can be attributed to the previous use of 0.2% glucose in the growth media used by Ref. [26], since *hyc* expression is repressed by glucose [36].

In order to maximize H_2_ production yield, it is of importance to understand whether conditions used to induce H_2_ production, such as decreasing pH, increase *hyc* expression or enhance the enzymatic activity. Our results show that a shift from pH 6.5 to 5.5, a shift from pH 5.5 to 6.5, or the addition of 5 mM benzoate did not affect H_2_ production, whereas expression of the large subunit of Hyd-3 increased as pH decreased from pH 7 to pH 5.5 (Fig. 3). Thus, the observed increase in H_2_ production is likely due to an increase in *hyc* expression [7], rather than a direct effect of external or internal pH on Hyd-3 activity.

Extreme-acid survival assays using aerobic cultures showed no hydrogenase-dependent acid resistance (Fig. 6). However, anaerobic cultures required Hyd-3 for survival at or below pH 2.5. The requirement for Hyd-3 increased as the pH decreased, showing a greater effect at pH 2 when compared to pH 2.5. Acid resistance systems are generally defined using aerobic cultures [7], [12], [15], [37] and Hyd-3 is the first reported mechanism that is necessary for anaerobic but not aerobic cultures.

The low-oxygen requirement for the acid resistance phenotype makes sense because Hyd-3 is only expressed anaerobically, controlled by the transcriptional activator FhlA [36], [38]; and under aerobic conditions Hyd-3 is inactive [28]. Nevertheless, other acid resistance systems are known to be co-induced by acid and anaerobiosis yet still show an acid resistance phenotype with aerobic cultures. For instance, arginine decarboxylase, *adiA*, is expressed when exposed to acid and low oxygen [7], [39]. The *adiA* system confers aerobic acid resistance [15], although *Salmonella typhimurium* requires anaerobic growth before extreme-acid exposure [22]. It is likely that greater attention to anoxic conditions will reveal new acid resistance components in *E. coli*.

## Materials and Methods

### Bacterial strains, media, and growth conditions

*E. coli* K-12 strain W3110 [40] was used as the wild-type strain in all experiments. Deletion alleles containing a kanamycin resistance insertion (Km^R^) were transduced from the Keio collection [41] into the wild-type strain by P1 phage transduction (Table 1).

**Table 1:** *E. coli* K-12 strains used in this study.

Strain	Genotype	Source
W3110	K-12 (F^−^ λ^−^)	[40]
JLS0920	W3110 *hyaB*::Km	This work
JLS0921	W3110 *hybC*::Km	This work
JLS0922	W3110 *hycE*::Km	This work
JLS0925	W3110 *hypF*::Km	This work

Cells were grown in LBK medium (10 g/L tryptone, 5 g/L yeast extract, and 7.45 g/L KCl) [7], [42]. Overnight growth medium was supplemented with kanamycin (25 µg/ml) for mutants. Media were buffered with 100 mM Homopiperazine-N,N'-bis-2-(ethanesulfonic acid) (HOMOPIPES), 2-(*N*-morpholino)ethanesulfonic acid (MES), 3-(*N-*morpholino)propanesulfonic acid (MOPS), or N-Tris(hydroxymethyl)methyl-3-aminopropanesulfonic acid (TAPS). The pH of the medium was adjusted as necessary using 5 M HCl or KOH. After growth, the pH of the medium was checked to ensure that pH was maintained within 0.2 units of that of the original uninoculated medium. Mutant and wild-type cells were streaked out on LBK-agar (supplemented with 25 µg/ml kanamycin for mutants) and stored at 4°C for up to a week.

### RNA isolation and real-time quantitative PCR

*E. coli* W3110 cultures were grown to mid-late log phase (OD_600_ = 0.4–0.5) as stated above in closed-cap anaerobic tubes at pH values from pH 5.5 to pH 7.0. Bacterial RNA from three independent cultures at each condition was stabilized using the cold 10% phenol-ethanol stop solution as previously described [7], [43] and isolated using the RNeasy Kit (Qiagen) followed by DNase treatment (Ambion).

Expression of *hycE* was quantified using real-time PCR using an ABI Prism7500 DNA analyzer (Applied Biosystems) as described previously [7]. The forward primer sequence was 5′ -GAAAACGCGATGGGTATTCAG - 3′ and the reverse primer sequence was 5′ - CAGAATGGCGCGGATCAT - 3′. The SYBR Green PCR One-Step Protocol (Applied Biosystems) was used, in which cDNA reverse transcription and PCR amplification occur in the same well. Nucleic acid concentrations were as follows: 0.1 nM forward primer, 0.1 nM reverse primer, and 50 ng target RNA. PCR cycling conditions were as follows: reverse transcription at 48°C for 30 min and 95°C for 10 min, 40 cycles of denaturation at 92°C for 15 s, and extension at 60°C for 1 min. The total RNA in each sample amplified was used as the basis to normalize individual gene expression profiles. Expression levels of the average of three technical replicates of each biological replicate are presented relative to the expression in the pH 7 anaerobic control.

### Acid resistance assays

The conditions for testing acid resistance (survival in extreme acid) were based on those described previously, with modifications [7], [12]. The percent of surviving cells was assessed using aerobic and anaerobic cultures. To test aerobic cultures, cells were grown to stationary phase without antibiotics (16–18 h, 37°C) in LBK 100 mM HOMOPIPES, pH 5 in culture tubes with a capacity 7.5 times the culture volume. The tubes were rotated vertically to ensure aeration (40 rpm). The overnight cultures were diluted 200-fold into unbuffered LBK at pH 2. Exposure tubes were incubated for 2 h with vertical rotary aeration (40 rpm) at 37°C. The initial cell density during extreme acid exposure was approximately 1×10^9^ CFU per ml. Following the 2 h exposure, the cultures were serially diluted and streaked onto LBK-agar plates. The overnight cultures were also serially diluted into LBK 100 mM MOPS, pH 7 and plated, representing the unexposed controls. Plates were incubated overnight at 37°C.

To measure acid survival of anaerobic cultures, cells were grown to stationary phase (16–18 h, 37°C) in LBK 100 mM MES, pH 5.5 in closed screw cap tubes. The tubes were rotated slowly end over end (8 rpm) to ensure that the cells distributed evenly throughout the medium [7], [44], [45]. The overnight cultures were diluted 200-fold into closed screw cap tubes with unbuffered LBK at pH 2.5 or pH 2. Exposure tubes were incubated for 2 h rotating end over end (8 rpm) at 37°C. The initial cell density during extreme acid exposure was approximately 3×10^8^ CFU per ml. Following the 2 h exposure, the cultures were serially diluted and plated. The overnight cultures were also serially diluted into LBK 100 mM MOPS, pH 7 and plated, representing the unexposed controls. Plates were incubated overnight at 37°C.

Survival rates were calculated as follows: the raw data were log_10_-transformed before taking the mean. The means were subtracted to get a log_10_ ratio, which roughly correlates to percent survival. The ratios are log_10_-transformed because survival data represent data points on an exponential death curve, and is thus expected to follow a log-normal distribution. All errors stated are standard error of the mean (SEM). Each experimental condition included six biological replicates from the same overnight culture.

### H_2_ production and consumption assays

To test H_2_ production and consumption, cells were grown to stationary phase (16–18 h, 37°C) in LBK buffered at the assay pH in closed screw-cap tubes rotating slowly (8 rpm) at 37°C. The cultures were diluted 100-fold into closed screw cap tubes with fresh media and grown to mid-late log phase (OD_600_ 0.4–0.5) rotating slowly (8 rpm) at 37°C. Cells were grown for 4 h (pH 5.5), 3 h (pH 6), 2.75 h (pH 6.5), 2.5 h (pH 7), and 2.5 h (pH 8). All strains grew at the same rate. Once grown to the appropriate OD_600_, 2 ml of the culture was used to fill a glass chamber (2 ml capacity). For H_2_ production assays the cultures were sparged with 100% N_2_ for 45 s to purge any existing H_2_ and maintain anoxic conditions. For H_2_ consumption assays the cultures were sparged with a 20% H_2_/80% N_2_ gas mixture for 90 s to saturate the solution with H_2_ and maintain anoxic conditions. The chamber was capped to seal the chamber from ambient air, and placed in a water bath (37°C) with a magnetic stir bar (200 rpm). H_2_ production and consumption were assayed for 6 minutes using a Clark-type electrode H_2_ micro-sensor system (Unisense; H_2_-MR-3306) [46]. Before the start of the assay, the electrode was incubated in LBK saturated with 20% H_2_/N_2_ for 1 hour, and then exposed to LBK saturated with 20% H_2_/N_2_ for three 10 minute intervals to ensure a consistent H_2_ reading. Each condition and strain included three biological replicates from the same overnight culture.

For the external pH shift assays, the conditions were the same as the H_2_ production assays except that the assay was conducted for 12 minutes, and KOH or HCl was added 6 minutes after the start of the assay to change the external pH from 5.5 to 6.5 or from 6.5 to 5.5. For the internal pH shift assays, the cells were again assayed for 12 minutes, and potassium benzoate was added 6 minutes after the start of the assay to expose the cells to a 5 mM final concentration.

The rate of H_2_ production and consumption was calculated based on the slope of H_2_ concentration (mV) versus time from 2–5 minutes and normalized to OD_600_. The first 2 minutes were used for sensor equilibration. The mV reading was converted to µM of H_2_ using a conversion factor of 0.0837 µM/mV. This factor was determined using the calibration procedure stated in the Unisense manual: a 2-ml sample of LBK was sparged with 20% H_2_/N_2_ and assayed at 37°C with a stir bar (200 rpm). The maximum mV reading was correlated to known values of saturated H_2_ concentration. All errors stated are standard error of the mean.
